# Effect of gadolinium-based nanoparticles on nuclear DNA damage and repair in glioblastoma tumor cells

**DOI:** 10.1186/s12951-016-0215-8

**Published:** 2016-07-28

**Authors:** Lenka Štefančíková, Sandrine Lacombe, Daniela Salado, Erika Porcel, Eva Pagáčová, Olivier Tillement, François Lux, Daniel Depeš, Stanislav Kozubek, Martin Falk

**Affiliations:** 1 Department of Cell Biology and Radiobiology, Institute of Biophysics of ASCR, Brno, Czech Republic; 2 Institute des Sciences Moléculaires d’Orsay (ISMO), Université Paris Sud 11, CNRS, Université Paris Saclay, Bât 351, 91405 Orsay Cedex, France; 3 Institut Lumière Matière, Université Claude Bernard Lyon 1, CNRS, 69622 Villeurbanne Cedex, France

**Keywords:** Radiosensitization, Nanomedicine, Gadolinium, Nanoparticles, DNA double-strand breaks, DNA repair, Radiotherapy, Theranostic

## Abstract

**Background:**

Tumor targeting of radiotherapy represents a great challenge. The addition of multimodal nanoparticles, such as 3 nm gadolinium-based nanoparticles (GdBNs), has been proposed as a promising strategy to amplify the effects of radiation in tumors and improve diagnostics using the same agents. This singular property named theranostic is a unique advantage of GdBNs. It has been established that the amplification of radiation effects by GdBNs appears due to fast electronic processes. However, the influence of these nanoparticles on cells is not yet understood. In particular, it remains dubious how nanoparticles activated by ionizing radiation interact with cells and their constituents. A crucial question remains open of whether damage to the nucleus is necessary for the radiosensitization exerted by GdBNs (and other nanoparticles).

**Methods:**

We studied the effect of GdBNs on the induction and repair of DNA double-strand breaks (DSBs) in the nuclear DNA of U87 tumor cells irradiated with γ-rays. For this purpose, we used currently the most sensitive method of DSBs detection based on high-resolution confocal fluorescence microscopy coupled with immunodetection of two independent DSBs markers.

**Results:**

We show that, in the conditions where GdBNs amplify radiation effects, they remain localized in the cytoplasm, i.e. do not penetrate into the nucleus. In addition, the presence of GdBNs in the cytoplasm neither increases induction of DSBs by γ-rays in the nuclear DNA nor affects their consequent repair.

**Conclusions:**

Our results suggest that the radiosensitization mediated by GdBNs is a cytoplasmic event that is independent of the nuclear DNA breakage, a phenomenon commonly accepted as the explanation of biological radiation effects. Considering our earlier recognized colocalization of GdBNs with the lysosomes and endosomes, we revolutionary hypothesize here about these organelles as potential targets for (some) nanoparticles. If confirmed, this finding of cytoplasmically determined radiosensitization opens new perspectives of using nano-radioenhancers to improve radiotherapy without escalating the risk of pathologies related to genetic damage.

## Background

Radiation-based therapies are used to treat half of cancer patients. Most common treatments based on highly penetrating MeV photons (X-rays and γ-rays) have the advantage of being non-invasive and applicable on inoperable tumors. However, the photon radiotherapy suffers from a serious disadvantage—it lacks tumor specificity. Photons induce damage all along their tracks, inflicting thus severe side effects in the healthy tissue. On the other hand, some tumors are resistant to high-energy photons. Therefore, a simultaneous enhancement of tumor selectivity and biological effectiveness of radiations is a long-lasting objective of cancer radiotherapy.

Nanoparticles (NPs) composed of high-Z atoms have been proposed as new nanodrugs able to improve both these desired aspects of radiation-based therapies (specificity and efficiency). Results obtained with various NPs showed that they can specifically increase radiosensitivity of tumor cells [1–4]. The use of nano-size agents that preferentially accumulate in the tumor (even passively due to the enhanced permeability and retention effect, EPR) [5, 6] may achieve the paradigm of local treatment of solid tumors. Among metal-based NPs, gold NPs have been widely used for diagnostics as contrast agents, and for therapy as nano-enhancers of radiation effects [7–11]. Gold NPs potentiate the effects of different photon beams, both in vivo and in vitro [1–4, 10, 12–14]. More recently, we have found [15, 16] that also platinum NPs enhance the effects of radiations, γ-rays as well as fast medical ions. Likewise, metal oxide nanoparticles are already on the market, currently evaluated in oncology clinical trials as compounds for tumor diagnostic and cancer treatment [17, 18].

An important step forward has been the development of gadolinium-based nanoparticles (GdBNs), which can act as multimodal agents and improve not only the therapeutic index of the treatment but also MRI performance (theranostics) [19, 20]. Due to its atomic mass (Z = 64), gadolinium is a good electron emitter, which is the property required to enhance the radiation effects. When applied in combination with both low and high-energy X-rays, γ-rays [21, 22], or fast ions [23], GdBNs significantly amplify radiation-induced cell killing, even in the case of U87 glioblastoma cells derived from a highly aggressive and radio-resistant human tumor [24, 25]. Concomitantly, GdBNs can serve as good contrast agents [19, 26] while they are rapidly eliminated from the organism by the kidneys, with no evidence of toxicity [24, 27–30].

GdBNs exert strong radiosensitizing effect on tumors [22–24, 31–33] when combined with several types of radiation of different energies (≥keV). For γ-rays used in this work, the radiosensitization appears due to prominent physical processes, namely the photoelectric and Compton effects, in dependence of the photon beam energy. The cascade of GdBNs-mediated processes resulting to cell radiosensitization starts with electron ‘showers’ emitted from nanoparticles upon irradiation and continues with water radiolysis producing free reactive oxygen species (ROS) [34, 35]. As these ROS are concentrated in nano-clusters, they induce complex nanosized bio-damages that are lethal for the cells [36, 37]. NPs thus increase the ionizing density (and damage) at the nanoscale, without influencing the macroscopic dose deposition [36, 38–40]. In accordance with this hypothesis, Burger and co-workers [41] showed that a high focal concentration of NPs is required to ensure an increased cellular inactivation by irradiated NPs. Also the local effect model (LEM) simulations suggested that the nanosized character of dose amplification is the key aspect of the ‘nanosensitization’ [38, 39].

Though the radio-enhancing effect of GdBNs has been clearly proved and explained in terms of physics, the structures and processes targeted by these (and other) nanoparticles in cells remain a subject to controversy. The nuclear DNA is logically the first suspect: it represents a critical cell structure and its damage by double strand breaks (DSBs) is commonly considered as the cause of radiation-induced cell death [42, 43]. Hence, it has been proposed that nanoparticles radiosensitize cells through amplifying the DSBs damage. However, several in vitro studies demonstrated [12, 13, 23, 32, 44, 45] that the radiosensitizers (metal complexes or NPs) are located in the cell cytoplasm. Stated in other words, NPs seem to amplify cell killing without entering the nucleus. As discussed below, these results open the question of whether secondary electrons only produced in close vicinity of cytoplasmically localized NPs may reach and damage to a sufficient extent the cell nucleus or whether cytoplasmic structures in closer proximity to NPs represent another (or even a more important) target for NPs-mediated radiosensitization.

Jones et al. [46] showed that also the dose enhancement mediated by NPs can spread as far as several micrometers. Leung et al. [47] reported that electrons can travel up to 3 μm or even 1 mm when activated by a 50 kVp and 6 MV source, respectively; this flying range is sufficient to reach the nuclear DNA. Thus, at least some electrons from NP-mediated electron showers might directly damage the nuclear DNA [41]. Whether this is sufficient to enhance cell killing remains a question.

Important evidence that the cytoplasmic damage may strongly influence the cell nucleus emerged from recent microbeam experiments. The group of Kevin Prise demonstrated that also cytoplasmically micro-irradiated cells develop 53BP1 protein foci—the markers of DNA DSBs—dispersed in the nucleus [48]. Moreover, these experiments revealed that the radiation damage to the cytoplasm can elicit 53BP1 foci formation both in directly exposed and bystander cells, independently of the dose and number of cells targeted. Hence, we can conclude that the cytoplasmic injury might also be followed by DNA damage with a corresponding biological response, though its kinetics for the pan-cellular and cytoplasmic irradiations differs.

The expansion of radiation damage from the cytoplasm to the nucleus is thus probably mediated by ROS [49]. In accordance, we can hypothesize that NPs might enhance the nuclear DNA damage by amplifying ROS production in the cytoplasm. In addition, disruption to protein transport and synthesis in the cytoplasm upon high radiation doses may slow down or even preclude DNA repair and further contribute to the cell killing by irradiated NPs. However, the information on the damage exerted by NPs to the nuclear DNA remains very limited and conflicting as available studies feature huge heterogeneity precluding the combination of results. This situation calls for further comprehensive analyses comparing the impact of physico-biological properties of various NPs and different treatment protocols on the radiosensitization processes.

In this work, together with providing a detailed information on the intracellular localization of GdBNs, we evaluated by currently the most sensitive method to detect DSBs how these nanoparticles influence the radiation damage introduced to the genomic DNA and how these lesions are consequently repaired during a long period of time post-irradiation (PI) in radioresistant U87 human glioblastoma cells. Our results represent new, direct and surprising evidence on the radiosensitizing mechanism of GdBNs: We demonstrate that this mechanism does not rely on the amplification of DSBs damage in the genomic DNA. Rather, based on our previous findings, we suppose that injury to the endosomes and lysosomes play a crucial role. These results may change the current dogma suspecting the nuclear DNA and/or mitochondria as the key targets for the nanoparticle-mediated radiosensitization.

## Methods

*Gadolinium-based nanoparticles (GdBNs)* were synthesised by the group of O. Tillement (LPCML, Lyon, France). Briefly, the GdBN consist of a polysiloxane core surrounded by gadolinium chelates covalently grafted on the inorganic matrix. The procedure of synthesis is detailed in Morlieras et al. [50] and Mignot et al. [27]. Briefly, the diameter of GdBNs was 3.0 ± 1.0 nm and their molecular mass 8.5 ± 1 kDa. These nanoparticles are stable, so they can be lyophilized and stored at 4 °C. For the analysis of DNA DSBs, label-free GdBNs were used. For the localization experiments by confocal microscopy, GdBNs were fluorescently labeled with Cyanine 5.5 (GdBNs-Cy5.5) as described elsewhere [50]. We have demonstrated earlier, by using different microscopy techniques [including synchrotron radiation deep ultraviolet microscopy (SR-DUV), transmission electron microscopy, and confocal microscopy], that labeling of GdBNs with cyanine 5.5 does not influence the nanoparticle localization [31].

### Cell culture

U87 cells grew (37 °C, 5 % CO_2_) in Dulbecco’s modified essential medium (Life Technologies) supplemented with 10 % heat-inactivated fetal calf serum (PAA), 100 U/ml penicillin (PAA), 100 μg/ml streptomycin (PAA), and 1 % NEAA (Life Technologies).

### Cell irradiation with γ-rays

U87 cells grown on microscopic slides (for DNA damage detection experiment) or in culture flasks (for the clonogenic survival experiment) were irradiated in culture medium at room temperature (RT) with 1 or 4 Gy of γ-rays (1 Gy/min), delivered by a ^60^Co irradiator (Chisostat, Chirana). During irradiation, the samples were kept in thermo-isolating boxes to prevent sample infection and temperature changes, and then immediately returned to the incubator (37 °C, 5 % CO_2_).

### Quantification of GdBN-mediated cell radiosensitization by clonogenic assay

Part of U87 cells followed incubation with 1 mM GdBNs for 1 h and consequently some samples were irradiated with 1 or 4 Gy of γ-rays as described above. The survival of cells was quantified by clonogenic assay and compared for non-irradiated and irradiated cells, in both cases either incubated or not incubated with GdBNs. After irradiation, cells were trypsinized and plated into 60 mm Petri dishes (Falcon 3002) at a density of 100 surviving cells per dish. The plating efficiency was 13 %. After 14 days of incubation, the colonies were fixed with 50 % methanol and stained with 1 % methylene blue. The colonies were counted manually by an experience examiner to determine the cell surviving fractions.

### Confocal microscopy studies of GdBNs localization

U87 cells were incubated with GdBNs labeled with Cy5.5 (GdBNs-Cy5.5) (1 mM) for 1, 6, and 16 h, respectively. Afterward, the cells were rinsed three times with 1× PBS and maintained in HBSS medium during the time of observation. The localization of GdBNs by confocal microscopy was performed with a LEICA SP5 confocal system, under constant temperature and CO_2_ levels (37 °C and 5 % CO_2_), at the Centre de Photonique Bio-Medical (CPBM), University Paris Sud, Orsay, France. GdBNs-Cy5.5 fluorescence was excited at 633 nm and the emission was detected in the 650–750 nm range. Images were recorded for three different z-positions (0.2 μm-thick confocal slices) for each cell. Transmission imaging was performed to visualize the size and shape of the cells and to discriminate between the nucleus and the cytoplasm. The fluorescence images obtained were merged with the transmission images by ImageJ software (Rasband, W.S., ImageJ, U. S. National Institutes of Health, Bethesda, Maryland, USA, http://www.imagej.nih.gov/ij/, 1997–2011) to determine the intracellular localization of GdBNs-Cy5.5. The same settings were used to perform fluorescence spectroscopy. The spectra for different cell compartments were registered together with random background (out-of-cell) values.

### Immunostaining of nuclear DSBs and their visualization by confocal microscopy

DSBs were detected in spatially (3D) fixed cells using a high-resolution confocal microscopy; the procedure was optimized by Falk et al. [51]. To maximize the sensitivity and fidelity of DSBs analyses, we took advantage of a dual fluorescence immunostaining to simultaneously visualize γH2AX and 53BP1 repair foci, the independent markers of nuclear DSBs [52, 53].

U87 cells were incubated with 1 mM GdBNs for 1, 6, and 24 h, respectively, and consequently some samples were irradiated with 1 or 4 Gy of γ-rays (1 Gy/min) as described. At the times post-irradiation (PI) of 5, 15, 30 min, 1, 2, 4, 8, and 24 h, the cells were spatially (3D) fixed with 4 % formaldehyde in 1X PBS for 10 min/RT, washed 3 times for 5 min each in 1X PBS, permeabilized with 0.2 % Triton X 100/PBS for 15 min/RT, and again washed 3 times for 5 min each in 1X PBS. Before the incubation with the primary antibodies (10 min RT and then overnight at 4 °C), the cells were blocked with 7 % inactivated fetal bovine serum +2 % bovine serum albumin/PBS for 30 min at RT.

Antibodies from two different hosts were used to simultaneously detect two DSBs markers in the same nuclei: anti-phospho-H2AX (serine 139) (mouse, monoclonal, dilution 1:500, Upstate Biotechnology) and anti-53BP1 (rabbit, polyclonal, dilution 1:500, Cell Signalling). Secondary antibodies, affinity purified FITC-conjugated donkey anti-mouse (diluted 1:200) and Cy3-conjugated donkey anti-rabbit (diluted 1:100) (both from Jackson Laboratory), were applied for 1 h in the dark at RT after the pre-incubation of slides with 5.5 % donkey serum/PBS for 30 min at RT. After washing 3 times for 5 min each in 1X PBS, cells were counterstained with 1 μM TOPRO-3 (Molecular Probes) in saline sodium citrate (2× SSC). Vectashield medium (Vector Laboratories) was used for the final mounting of slides.

Forty *z*-stacks, acquired at 0.2 μm steps, were recorded (at IBP ASCR Brno, CR) in three separate spectral channels by the confocal microscope Leica SP5 (Leica Microsystems) and an automated Leica DM RXA fluorescence microscope equipped with a Nipkow disk (Jokogawa, Japan) for confocal imaging (in detail described in Kozubek et al. [54, 55]). The visualization and analysis of the 3D images were performed using the *Aquarium software* [56], *3D image viewer* [56], and *ImageJ software* (Rasband, W.S., ImageJ, U. S. National Institutes of Health, Bethesda, MD, USA, http://www.imagej.nih.gov/ij/, 1997–2011). Fifty to 100 cells were analyzed for each treatment and period of time PI. This approach allowed us to analyze (a) the initial induction of DSBs immediately after the irradiation (5 min PI), (b) the repair kinetics of these lesions during a long period of time post-irradiation (up to 24 h), and (c) the persistence of unrepaired DSBs at late times PI (8 and 24 h). The representative maximum images composed of 40 confocal 0.2 µm thick slices are shown in Figs. 3, 4, 5 and 6.

#### Data analysis

The SigmaPlot 12.5 (Systat Software Inc.) has been used for data analysis. The Mann–Whitney Rank Sum Test was employed to compare at all the particular periods of time PI the distributions of DSBs (γH2AX/53BP1 foci) numbers per nucleus in untreated control cells and cells incubated with 1 mM GdBNs for 1 h. The relevant P values are indicated in Tables 1 and 2. Around 100 nuclei were quantified in each single experiment. To mutually compare the distributions of DSBs for untreated controls and cells incubated with GdBNs for 1, 6, and 24 h, respectively, the Kruskal–Wallis One Way Analysis of Variance on Ranks (a non-parametric equivalent of the one-way analysis of variance, ANOVA) was applied; the corresponding P values are shown in Table 3.

**Table 1:** Effect of GdBNs on DSBs quantity in irradiated (1 Gy) U87 cells

	NI	5 min	15 min	30 min	1 h	2 h	4 h	8 h
U87	1.4	18.1	21.1	19.7	14.9	8.7	5.5	3.1
U87 + GdBNs	1.2	16.7	19.3	17.5	15.7	8.6	5.0	2.9
P	0.533	0.111	0.083	0.096	0.379	0.970	0.485	0.241

**Table 2:** Effect of GdBNs on DSBs quantity in irradiated (4 Gy) U87 cells

	NI	1 h	4 h	8 h	24 h
U87	1.9	42.4	20.6	12.2	5.0
U87 + GdBNs	2.0	42.9	20.1	12.3	4.9
P	0.059	0.731	0.916	0.350	0.686

**Table 3:** Effect of incubation times with GdBNs on DSBs quantity in irradiated (1 Gy) U87 cells

	NI	5 min	1 h	4 h	24 h
U87	1.6	12.7	12.3	4.9	2.2
U87 + GdBNs 1 h	1.6	14.4	12.7	5.8	
U87 + GdBNs 6 h	1.5	11.3	12.5	5.1	1.8
U87 + GdBNs 24 h		12.3	11.9	6.0	2.0
P	0.433	0.091	0.647	0.328	0.699

In Figs. 3, 4 and 5, the data are displayed in the form of box graphs showing the distributions of DSBs foci per nucleus. The boxes include 50 % of the values (25th to 75th percentile) centred on the median (the horizontal line through the box). The mean values are represented by the squares within the boxes. The vertical lines begin at the 5th percentile and end at the 95th percentile. Representative nuclei for each time point PI are shown above the respective box.

## Results

### Uptake and localization of GdBNs in U87 cells

**Fig. 1:**
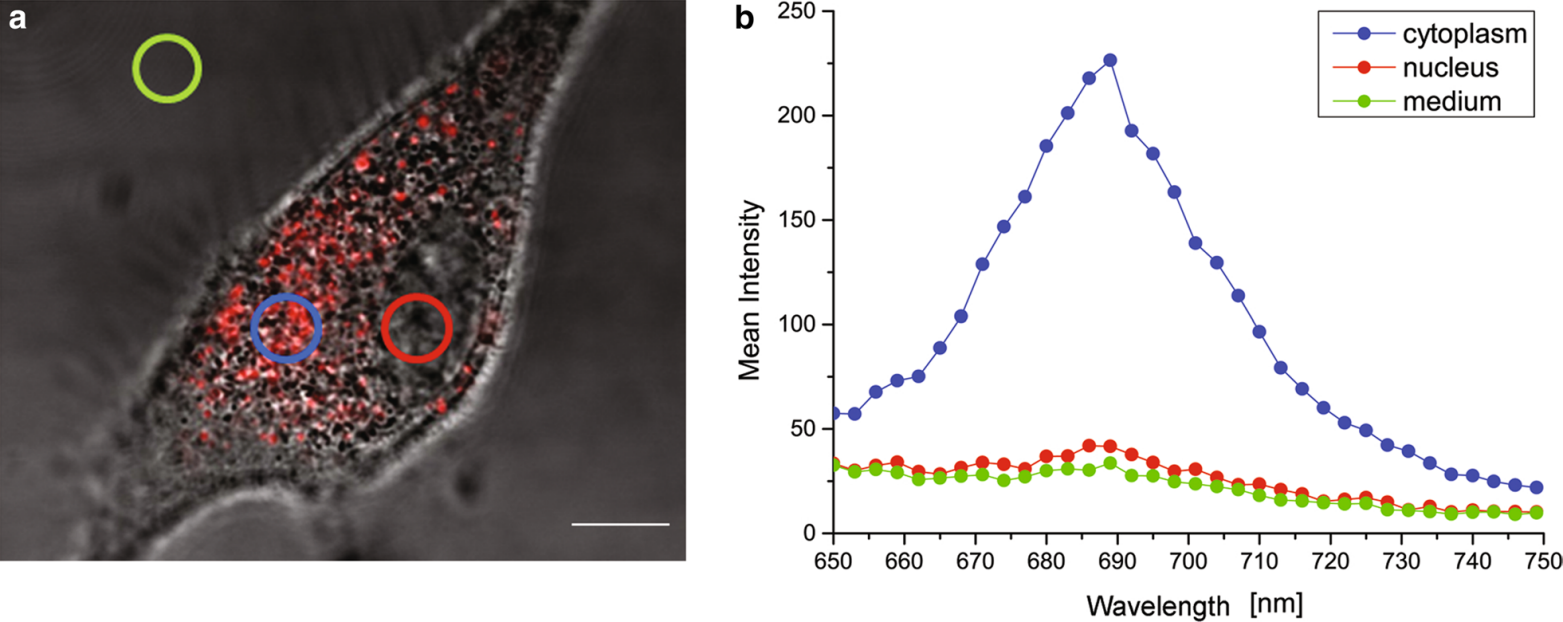
Localization of GdBNs-Cy5.5 nanoparticles in U87 cell. **a** Correlative fluorescence confocal image and transmission light image of U87 cell with internalized GdBNs-Cy5.5 (*red*) at the end of 16 h-long observation. The *scale bar* equals to 10 µm. The *circles* represent the regions of fluorescence spectroscopy measurements, cytoplasm (*blue*), nucleus (*red*), and plain medium (*green*). **b** Fluorescence emission spectra of the three regions

First, we analyzed by confocal microscopy the cellular uptake and localization of 1 mM GdBNs in U87 cells during 16 h-long period of observation. Figure 1a shows exclusively cytoplasmic localization of GdBNs (labelled with Cy5.5) as demonstrated by ‘correlative’ transmission light images and confocal fluorescence images. To further probe the intracellular distribution of GdBNs, we completed confocal microscopy by fluorescence spectroscopy (Fig. 1b) of the regions of interest located in (a) the nucleus, (b) the cytoplasm, and (c) the extracellular space (plain medium). The spectra measured in the cytoplasm displayed an intensive peak at λ = 688 nm, which corresponds to the fluorescence of GdBNs labelled with Cyanine 5.5. This peak was clearly absent in the spectra obtained inside the nucleus or outside the cells. Both microscopy and spectrometry thus confirmed absence of GdBNs in the cell nucleus.

**Fig. 2:**
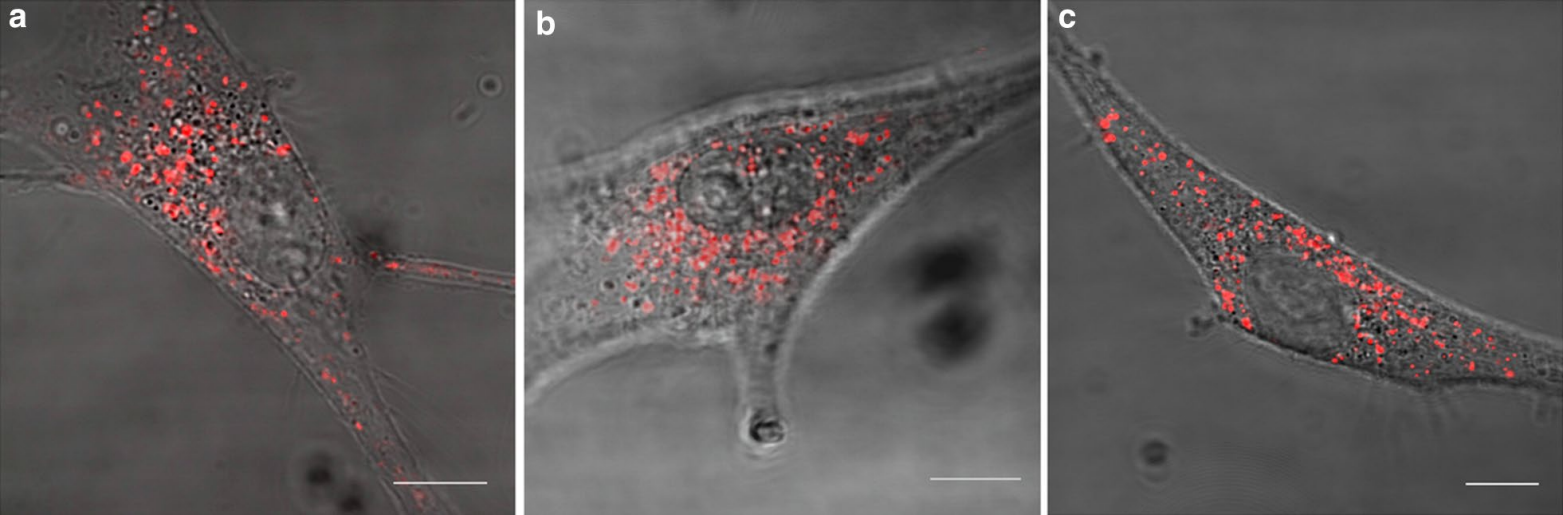
Localization of GdBNs-Cy5.5 nanoparticles in U87 cells as a function of the incubation time. Correlative fluorescence confocal images and transmission light images of U87 cells incubated with 1 mM GdBNs-Cy5.5 (*red*) for three different incubation times: **a**—1 h, **b**—6 h and **c**—16 h. *Scale bars* equal to 10 µm

Next, we compared the uptake and localization of GdBNs in U87 cells also for two shorter incubation periods: 1 and 6 h. As demonstrated by Fig. 2, GdBNs were already internalized after 1 h incubation and longer incubation times of 6 and 16 h (see also Fig. 1) had no influence on GdBNs uptake efficacy. For all the periods of time studied, GdBNs were localized in the cytoplasm of U87 cells without penetrating into the nucleus. In contrast to the situation described for SQ20B cells by Miladi and coworkers [33], we observed no clustering of NPs on the cytoplasmic membrane.

### Effect of GdBNs on nuclear DNA damage in U87 cells

We investigated whether GdBNs alone or in combination with irradiation cause the nuclear DNA damage and/or influence repair of these lesions. We focused on the DNA double strand breaks (DSBs; visualized as γH2AX foci co-localizing with 53BP1 foci) that represent the most important type of DNA damage.

#### Effect of GdBNs on nuclear DNA in non-irradiated cells (nanoparticle genotoxicity)

Biological toxicity of nanoparticles represents a critical issue in therapy. We demonstrated earlier [31] that GdBNs used in this study are not toxic and neither the survival nor the division of cells. However, several authors reported that the silver [57–59] and gold nanoparticles enhance the levels of γH2AX [60] and the oxidative stress [61], both in normal and cancer cells. Hence, we further investigated the effect of our GdBNs on the DNA integrity in U87 cells without irradiation.

**Fig. 3:**
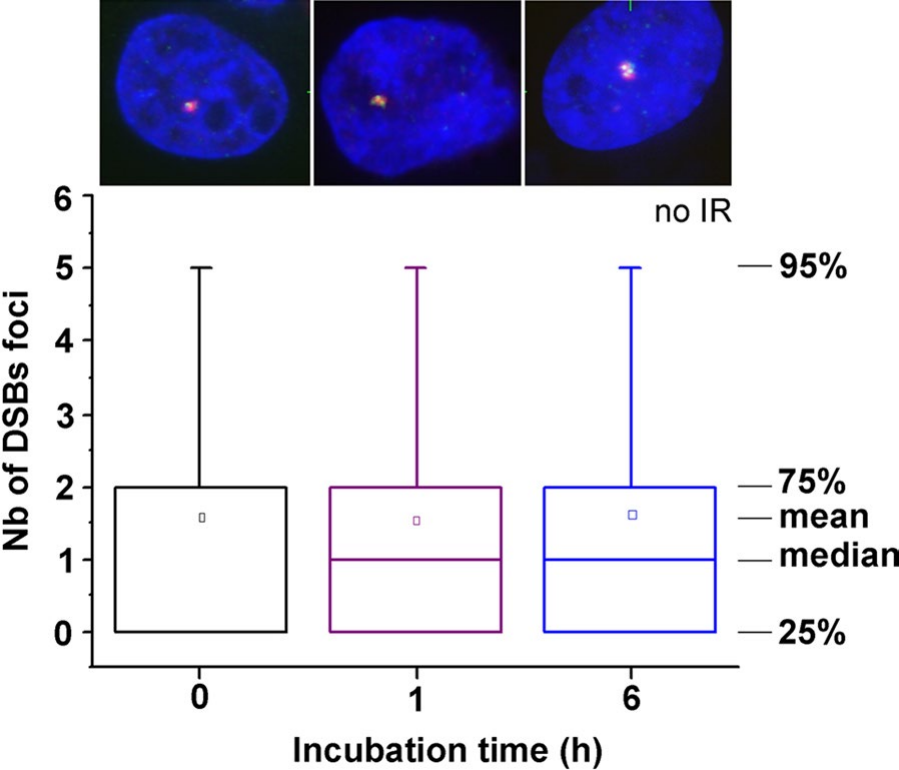
Effect of GdBNs on DSBs formation in non-irradiated U87 cells. Distribution of DSBs foci numbers are compared for non-irradiated U87 cells never incubated with GdBNs (*black*) and incubated with 1 mM GdBNs for 1 h (*purple*) and 6 h (*blue*). The respective cell nuclei are shown as the maximum images (composed of 40 confocal slices 0.2 μm-thick) with 3D projections; γH2AX—*green*, 53BP1—*red*, chromatin—artificially *blue*

Figure 3 compares the distribution of γH2AX/53BP1 (DSBs) foci numbers in U87 cells never incubated with GdBNs (control cells) and incubated with 1 mM GdBNs for 1 and 6 h, respectively. For both the periods of time, without irradiation, the cell treatment with GdBNs had no effect on the number of DSBs detected. The average values from two independent experiments were 1.6 DSBs/nucleus for untreated cells, 1.5 DSBs/nucleus for cells incubated with GdBNs for 1 h, and 1.6 DSBs/nucleus for cells incubated with GdBNs for 6 h. Hence, GdBNs of parameters used in this work are not genotoxic by themselves.

#### Effect of GdBNs on nuclear DNA DSBs induction and repair in irradiated U87 cells

**Fig. 4:**
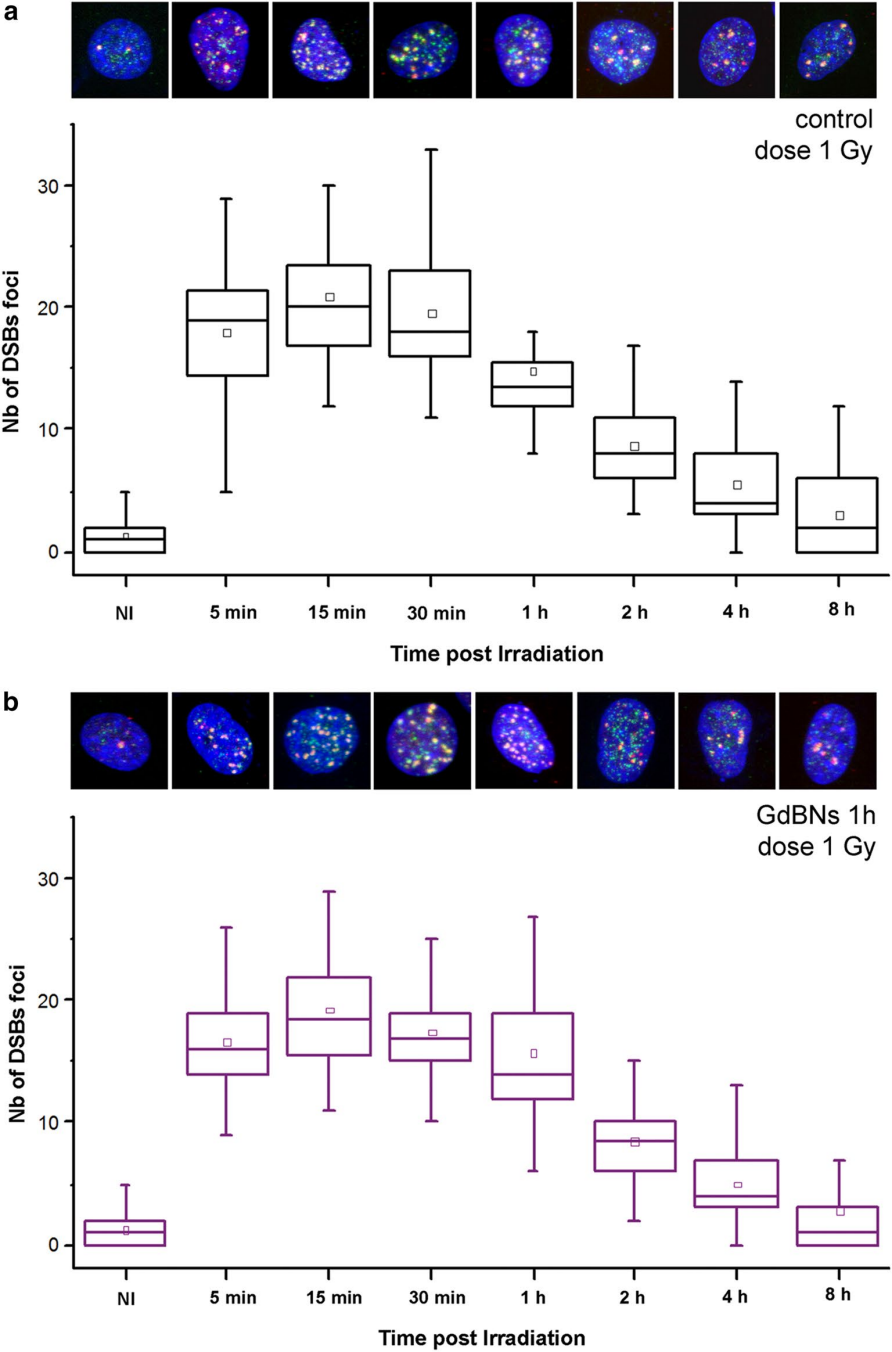
Effect of GdBNs on DSBs formation and repair in irradiated (1 Gy) U87 cells. Distribution of DSBs foci numbers are compared for irradiated U87 cells **a** never incubated with GdBNs and **b** incubated with 1 mM GdBNs for 1 h. Non-irradiated controls are indicated as NI. The respective maximum images of representative nuclei for each period of time PI are shown above: γH2AX—*green*, 53BP1—*red*, chromatin—artificially *blue*

In the next step, we studied how cytoplasmic GdBNs influence the extent and reparability of DSBs introduced to the nuclear DNA by irradiation with two different doses of γ-rays, 1 and 4 Gy, respectively. The application of high resolution confocal immuno-fluorescence microscopy with two independent DSBs markers (γH2AX and 53BP1) allowed us to precisely analyze the extent of DSBs induction in intact cells as early as 5 min post-irradiation (PI). Consequently, we evaluated the repair of DSBs in terns of γH2AX/53BP1 foci disappearance over 8 h-long period of time PI; this period is sufficient to repair the majority of DSBs and allows considering effects of the two main DSBs repair pathways, NHEJ—non-homologous end-joining, and HR—homologous recombination. To follow both the kinetics and the final efficiency of DSBs repair, we scored γH2AX/53BP1 foci in 7 time points PI. The results for U87 cells irradiated with 1 Gy of γ-rays and incubated or not incubated with 1 mM GdBNs for 1 h are summarized in Fig. 4 and Table 1.

Average numbers of DSBs foci per nucleus at indicated periods of time PI are compared for U87 cells irradiated with 1 Gy of γ-rays in absence or presence of 1 mM GdBNs (1 h incubation). Non-irradiated control cells (NI) are included. P values indicate the significance of differences between cells untreated and treated with GdBNs, respectively.

For all the periods of time PI, we observed comparable mean/median numbers of γH2AX/53BP1 foci per nucleus between the U87 cells incubated with GdBNs and the untreated controls. These results show that both the extent of DSBs induction measured at 5 min PI and the kinetics of DSBs repair between 5 min PI and 8 h PI are not affected by GdBNs present in the cytoplasm of irradiated U87 cells.

**Fig. 5:**
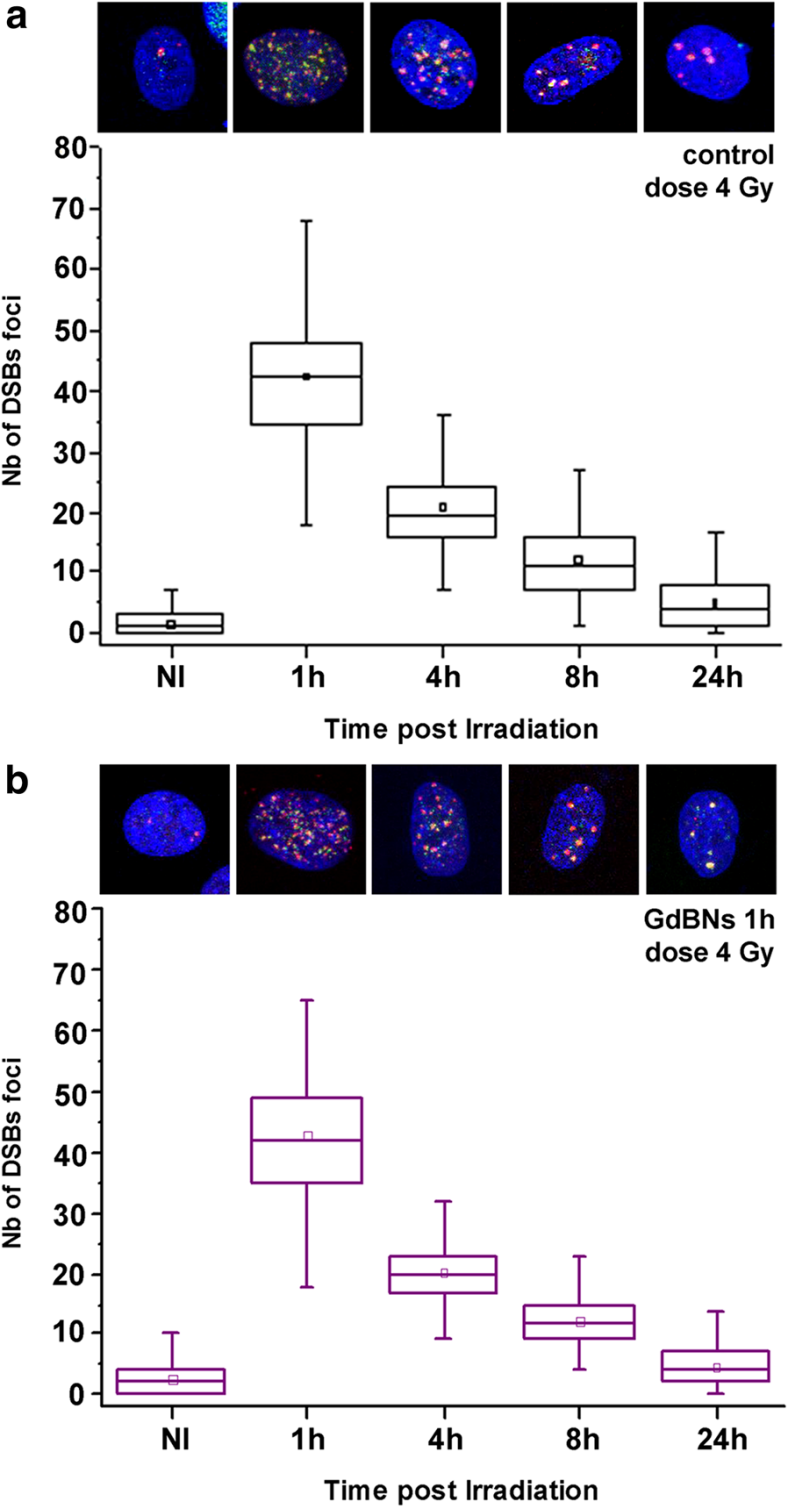
Effect of GdBNs on DSBs formation and repair in irradiated (4 Gy) U87 cells. Distribution of DSBs foci in U87 cells never incubated with GdBNs (**a**) and incubated with 1 mM GdBNs for 1 h (**b**). Non-irradiated controls (NI) are also compared. The respective maximum images of representative nuclei for each period of time PI are shown above: γH2AX—*green*, 53BP1—*red*, chromatin—artificially *blue*

To check whether these conclusions hold also for higher radiation doses, we repeated the above described experiments also with 4 Gy irradiation. This dose also ensures more extensive DNA damage which in turn allows recognition of smaller differences between the compared samples. U87 cells were again incubated with 1 mM GdBNs for 1 h; however, this time we have focused on less time-points (1, 4, 8 and 24 h) but dispersed along a period of time extended up to 24 h PI. Indicated time-points were selected as they allowed us to estimate: (1) the extent of DSBs induction (since the numbers of γH2AX/53BP1 foci at 1 h PI still approach the maximum values), (2) the efficiency of NHEJ and HR repair pathways (4/8 h PI, respectively), and (3) also the extent of DSBs that are repaired only with difficulty (and persist in nuclei 24 h PI, when the repair process are usually accomplished even for 4 Gy and higher dose γ-irradiations). The results are summarized in Fig. 5 and Table 2. As for 1 Gy, we found only insignificant differences between the mean/median γH2AX/53BP1 (DSBs) foci numbers in cells incubated or not incubated with GdBNs.

Altogether, these results indicate that our GdBNs (1 mM)—alone or in combination with irradiation—do not affect nuclear DNA. GdBNs of defined parameters influenced neither the induction of DSBs nor the kinetics and efficiency of their repair. Based on these results, we conclude that GdBNs may amplify radiation-induced cell killing through effects independent on the nuclear DNA.

Average numbers of DSBs foci per nucleus at different periods of time PI are compared for U87 cells irradiated with 4 Gy of γ-rays in absence or presence of 1 mM GdBNs (1 h incubation). Non-irradiated control cells (NI) are also included. P values indicate the significance of differences between cells treated and untreated with GdBNs.

### Influence of the incubation time with GdBNs on DSBs foci induction by γ-rays and their repair

As reviewed in Sancey et al. [24], available studies on GdBNs used different nanoparticle incubation times. At the same time, several reports with gold [62] and gadolinium [32] NPs demonstrated that this experimental parameter has a significant effect on NPs concentration and distribution in the cells. This makes comparisons and interpretations of results problematic. Thus, we investigated how different times of incubation with our GdBNs influence the induction of DSBs by γ-rays and repair of these lesions.

**Fig. 6:**
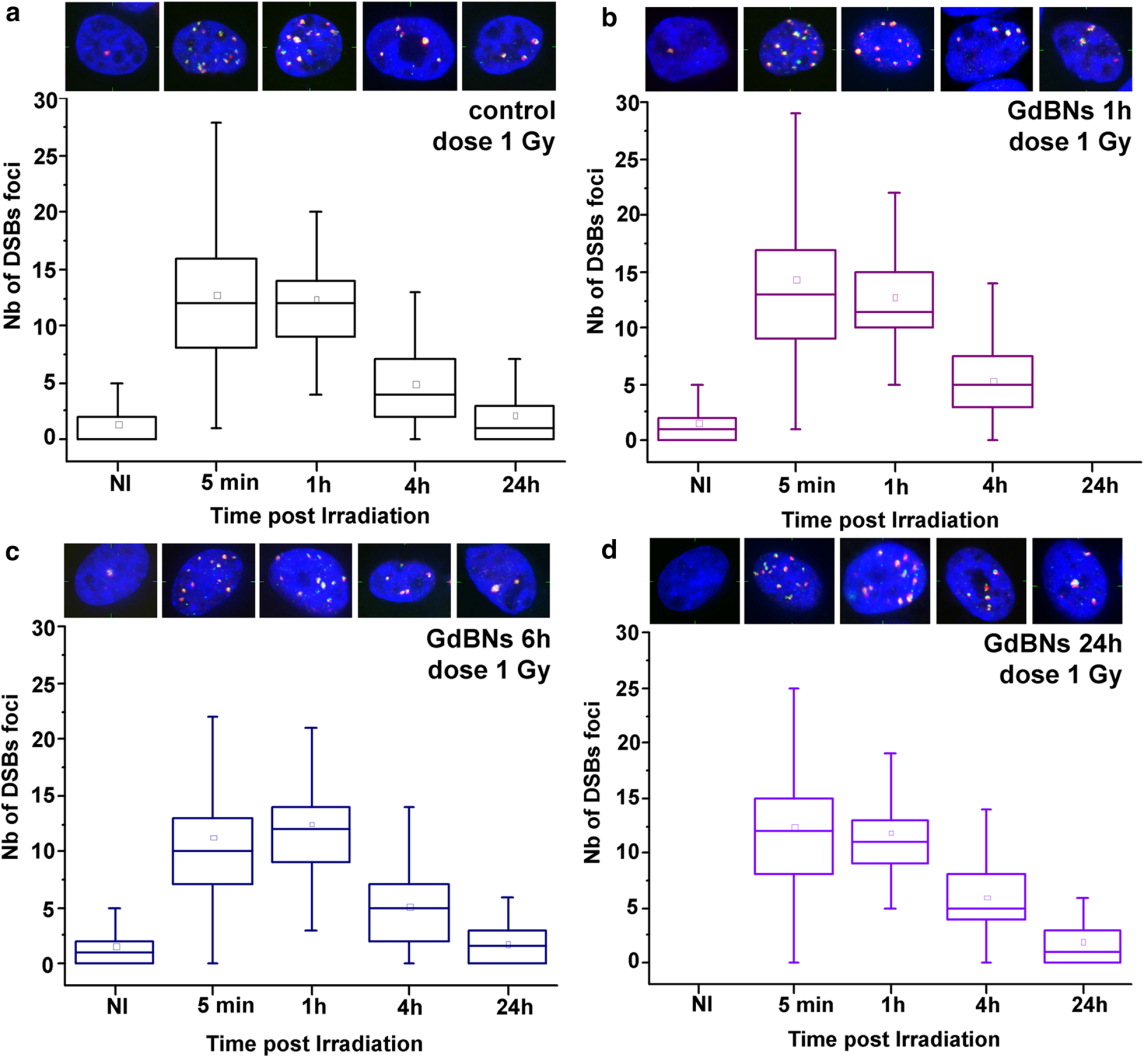
Effect of incubation times with GdBNs on DSBs formation and repair in irradiated U87 cells. Distributions of DSBs foci numbers are compared for U87 cells irradiated with 1 Gy of γ-rays and never incubated with GdBNs (**a**) or incubated with 1 mM GdBNs for 1 h (**b**), 6 h (**c**) and 24 h (**d**). Non-irradiated controls (NI) are also compared. The respective maximum images of representative nuclei are shown above: γH2AX—*green*, 53BP1—*red*, chromatin—*blue*

As in the previous experiments, we quantified γH2AX/53BP1 (DSBs) foci in U87 cells incubated with 1 mM GdBNs and exposed to 1 Gy of γ-rays. The results for 0, 1, 6, or 24 h-long incubations followed by DSBs quantification at 5 min, 1, 4, and 24 h PI, respectively, are presented in Fig. 6 and Table 3. Evidently, the prolonged incubations increased neither the induction of DSBs lesions nor delayed their repair. These results thus agree with our observation presented in Fig. 2 that GdBNs do not penetrate in the nucleus even at longer incubation times.

Average numbers of DSBs foci per nucleus in different periods of time PI are compared for U87 cells irradiated with 1 Gy of γ-rays in absence or presence of GdBNs, applied for 1, 6 or 24 h prior to irradiation. Non-irradiated control cells (NI) are also included. P values indicate the significance of differences between samples for each period of time PI.

### Radiosensitizing effect exerted by irradiated GdBNs

**Fig. 7:**
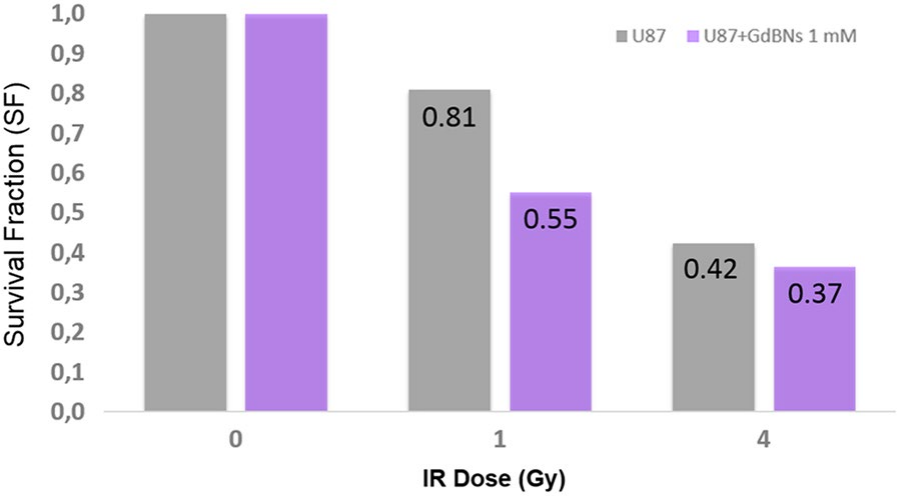
Effect of GdBNs on clonogenic survival fraction of U87 cells. Surviving fractions of U87 cells never incubated with GdBNs (*grey*) or in the U87 incubated with 1 mM GdBNs for 1 h (*purple*) and irradiated by 1 and 4 Gy of γ-rays (^60^Co), respectively. Non-irradiated controls (dose 0) were normalized to 1

Recently, we have shown that GdBNs of the parameters and concentration used in this work (1 mM) exert a substantial radiosensitizing effect in CHO cells irradiated with He2+ or C6+ high energy ions [23]. In our previous work, we have also confirmed the radiosensitizing effect of these GdBNs in U87 cells irradiated with γ-rays [31]. However, a controversy exists in the literature on the radiosensitizing efficiency of higher (about >1 mM) GdBN concentrations. Hence, we confirmed here by clonogenic assay the effects of 1 mM GdBNs on the cell vitality and proliferation potential. Figure 7 shows significantly lower clonogenic survival of U87 cells in presence of GdBNs at doses 1 and 4 Gy, respectively; the non-irradiated controls are also included. Though the NP-mediated radiosensitization is less prominent at 4 Gy as compared with 1 Gy dose (see Discussion), these results unequivocally confirm the presence of the radiosensitizing effect upon the conditions used in this work. Therefore, missing effects of GdBNs on nuclear DNA damage and repair support the idea of cell radiosensitization by GdBNs that originates in the cytoplasm, instead of reflecting the absence of the radiosensitizing effect.

## Discussion

To our best knowledge, there are only few other reports on the effects of nanoparticles on DSBs formation and/or repair upon irradiation. In this work, we show that nanoparticles irradiated in the cytoplasm can potentiate radiation-induced cell killing without a need to penetrate into the cell nucleus and damage DNA. This conclusion might be surprising concerning the fact that the nuclear DNA is undoubtedly the most important and, at the same time, fragile structure in the cell. For a long time, a direct damage to DNA has been assumed as the key event starting the cascade of reactions mediating the cell response to irradiation [63]. However, microbeam irradiations only restricted to specific cellular subcompartments [49, 63, 64] clearly demonstrated that DNA can be damaged even without being directly hit; the cytoplasmic and also the extracellular irradiation triggered similar DNA damage and associated important cellular pathways as the dose deposited in the nucleus [65–67]. Moreover, several studies suggested that damage to the mitochondria or cell membrane markedly contribute to the cytotoxic effect of radiations [68]. Therefore, the whole cell, rather than DNA only, should be considered a sensor of radiation exposure [64].

The only cytoplasmic organelles containing DNA in human cells are the mitochondria. As the ‘energy generators’, the mitochondria are vital for the cell. However, our previous colocalization studies [31] excluded the possibility that GdBNs localize into (or close to) these organelles. On the other hand, we have revealed that GdBNs of parameters used in this work colocalize with the lysosomes in U87 cells [31]. In the light of our further findings presented here, i.e. that NPs affect neither the damage nor the repair of the nuclear DNA, we propose a provoking hypothesis that the radiosensitization mediated by our GdBNs is triggered by damage to the lysosomes and endosomes and potentially other cytoplasmic organelles in their proximity.

Several reports [69–72] revealed only recently that the lysosomes, these still mysterious organelles, play an important role in the initiation of the cell death signalling (reviewed e.g. in [72, 73]), regulation of the cell cycle [73] and energy metabolism (reviewed e.g. in [74]). Already a moderate lysosomal rupture forces the cell to apoptosis while more pronounced lysosomal leak results in necrosis without caspases activation [72, 73, 75]. Though we currently run experiments on this topic, we cannot provide a direct evidence for the lysosome damage mediated by our GdBNs (since these NPs are no more available). Nevertheless, Heid et al. [76] recently demonstrated that release of mitochondrial ROS subsequently leads to the lysosomal membrane permeabilization (LMP). Hence, we can legitimately suppose that huge amounts of ROS produced by irradiated NPs in the lysosomes can easily disintegrate the membranes in their substantial fraction, with the already described consequences for the cell.

In accordance with fundamental changes in the long-accepted paradigm on the role of the cytoplasm in the cell response to radiation, our results seem to disclose new important features of the mechanism by which GdBNs exert their radiosensitizing effect; however, the details and complexity of NPs-mediated cellular radiosensitization still remain a mystery. We speculate here about new molecular targets for NPs, other than the nuclear or mitochondrial DNA; this offers a broad scale of new opportunities for much safer therapeutic attacks on cancer cells. Indeed, many survival attributes of neoplastic cells are determined by extra-nuclear structures and processes, including mitochondrial and lysosomal proteins involved in (anti)apoptotic, cell cycle, and cell damage signaling pathways.

Our preliminary experiments (results not shown) with other cell types and nanoparticle types of the similar size as GdBNs used in this work suggest that the conclusions (the cytoplasm damage-based radiosensitization, no escalation of DNA damage and no DNA repair inhibition), postulated in the above paragraphs for U87 cells and GdBNs, could be more generally valid.

As discussed later in more detail, many key factors may determine intracellular localization and distribution of NPs and, in turn, the extent and the mechanism of radiosensitization. It is, for instance, the size of NPs, which also dictates the effectivity of NPs intake and exclusion by the cells (e.g. Moser et al. [77]). The NPs concentration and composition as well have been reported to influence distribution of NPs in cells [32, 33] with a significant impact on the radiosensitization intensity and, perhaps, its mechanism (see later). Finally, as also demonstrated by our results, the radiation dose seems to be unimportant concerning the mechanism of NP-mediated cell radiosensitization (physical processes of NPs activation and damage introduction to biomolecules are still the same) but the contribution of NP-mediated effects to cell killing by irradiation depends on the dose (see Fig. 7). As soon as the radiation dose is high enough to activate NPs to an extent sufficient for damaging cytoplasmic organelles (lysosomes or other) in NPs proximity, additional dose escalation could not be expected to further increase the radiosensitization (while radiation damage to DNA still grows with the dose). Hence, for higher doses, the additional value of radiosensitization to therapy relatively decreases (see Fig. 7). These results may also explain why some authors, working with higher radiation doses, did not observed radiosensitizing effect of 1 mM GdBNs [22]. To conclude, our results are not limited to the cell type, nanoparticle type and conditions used herein; however, they should be generalized only with caution since our understanding to biological processes that take place in cells after the cytoplasmic irradiation, especially if NPs are present, is still very limited. Systematic studies in living cells are also necessary to understand how the key physical and biological factors mutually interact in providing the final radiosensitizing effect.

In addition to the efficiency, the genotoxicity of NPs represents another crucial issue in the context of therapy. It has been already demonstrated for cells loaded with silver NPs [58, 59] that even cytoplasmically localized NPs can induce DSBs without being irradiated. Preferentially sequestered in tumors though, NPs thus seem to undesirably damage also normal cells. By contrast, we showed here by currently the most sensitive method to detect DSBs that GdBNs of our parameters and under conditions used in this work are not genotoxic. This points to the importance of careful studies on the genotoxicity of each particular NPs type. Taken together, our results open new optimistic horizons for further development of efficient but safe NPs-based therapies of malignant and also non-malignant diseases; however each individual NPs type should be carefully characterized before being used in clinical practice, both in terms of its physical properties and biological activity.

There are only few studies our results can be compared with. In agreement with the present work, Jain et al. [2] demonstrated that 1.9 nm gold NPs neither enhance radiation-induced DSBs formation nor inhibit DNA repair in MDA-MB-231 breast cancer cells irradiated with MV electrons. On the other hand, Chithrani et al. [3] observed an increase of DSBs induction in HeLa cells incubated with 50 nm citrate-coated gold NPs irradiated with 6 MV photons. Similarly, Berbeco and co-workers [78] described a significant increase in DNA damage for 50 nm gold NPs in HeLa cells when activated by clinical MV photon beams. Finally, Zhu and co-workers [79] showed augmentation of DNA damage for megavoltage X-rays (6 MeV) and gold NPs in the size range of 20–74 nm (HepG2 cells). Taken together, it seems that bigger gold NPs enhance the DNA damage while the smaller NPs do not. Contradictory effects of NPs on DNA might be thus, at least partially, explained by their size. Indeed, for physical reasons, nanoparticles of 50 nm in diameter provide stronger radioenhancing effect than their smaller variants [3, 41]. However, Mowat et al. [22] and Miladi et al. [33] evidenced a significant rise in the nuclear DNA damage when they irradiated U87 or SQ20B cells in presence of GdBNs as small as 3 nm.

In the context of present article, Mowat’s work [22] is particularly interesting since the authors used similar experimental design (U87 cells, gadolinium-based NPs, and γ-ray irradiation) but came to different conclusions. The difference between our results and those of Mowat and coauthors [22] might reflect several factual and/or experimental factors:

1. Nanoparticles design: The design of GdBNs used in the present and Mowat’s work [22] is different. We cannot exclude that differently designed NPs (gadolinium oxide core surrounded by polysiloxane shell vs. polysiloxane core surrounded by gadolinium chelates covalently grafted on the inorganic matrix), though composed of the same material, behave differently in cells and increase cell killing by various biological mechanisms.
2. Nanoparticles concentration and intracellular localization: Rima et al. [32] revealed by transmission electron microscopy that the number per cell of vacuoles containing GdBNs as well as the average size of these vacuoles increase with GdBNs concentration up to 0.6 mM; however, for concentrations up to 2 mM the average vacuole size still increases but their number per cell decreases. Confirming a functional importance of these findings, another work demonstrated that the size of NPs clusters is more relevant parameter determining the radiosensitizing effect of NPs than their intracellular concentration as the whole [32]. The concentration effects were also mentioned in a recent article of Miladi et al. [33] where GdBNs (labelled with Cy5.5) started to cluster on the membrane of SQ20B cells when higher (≥0.8 mM) NP concentrations were used. By contrast, with 1 mM GdBNs, we observed an intensive nanoparticle uptake by U87 cells without any signs of their accumulation on the membrane. This strictly cytoplasmic residence of 1 mM GdBNs agrees with our previous results in CHO cells [23] obtained with NPs of the same parameters.
  Along with influencing the NPs intracellular distribution, the concentration of NPs seems to affect the mechanism of radiosensitization and the final radiosensitizing effect. The experiments have been performed particularly using low gadolinium concentrations ranging from 0.1 to 1 mM (see Table 3 in the review paper [24]). In two different cancer cell lines, U87 (glioblastoma cell line) and SQ20B (squamous cell carcinoma cell line), the moderate gadolinium concentrations (0.4–0.7 mM) potentiated the effects of radiation most efficiently in in vitro conditions [22, 28]. Mowat and co-workers [22] showed that while 0.5 mM GdBNs substantially enhance radiation-induced DSBs foci formation as quantified by comet assay, this effect is absent or only minor for higher (1 and 2 mM) GdBNs concentrations. In other studies [22, 29], enhanced DNA damage as monitored by γH2AX levels appeared for the concentrations between 0.4 and 0.6 mM but not for the concentration of 2 mM [22]. In accordance, Rima et al. [32] found that the quantity of gadolinium in U87 cells and SQ20B cells increases almost linearly with the GdBNs concentration but the cell killing by irradiation peaks at the concentration of 0.6 mM and almost disappears at 1 mM. In contrary to the above mentioned studies, Porcel et al. [23] demonstrated significant radiosensitization of CHO cells exposed to He2+ and C6+ high energy ions, respectively, in presence of 1 mM GdBNs of the same parameters as used in the present work. The extent of U87 cell radiosensitization by 1 mM GdBNs after irradiation with 1 or 4 Gy of gamma rays is quantified in Fig. 7 and is obvious. Even small differences in the GdBNs concentration (in combination with other factors) may thus dramatically change the radiosensitizing effect; the concentrations around 1 mM seem to be most controversial in this respect. Hence, by choosing 1 mM GdBNs, we aimed to complete the mentioned studies and make another step towards our better understanding of the radiosensitization mediated by these NPs.
3. Nanoparticle surface modifications and labeling: Mowat et al. [22] used GdBNs conjugated with FITC for their DSBs studies. While we have proved consistently that the intracellular behavior of free GdBNs and GdBNs labeled with Cy5.5 does not differ [31], it is possible that FITC influences the uptake, distribution, and intracellular localization of NPs. This becomes evident when one compares the localization of GdBNs conjugated with Cy5.5 [31] and those with FITC [22]. Considering this risk of experimental artifacts, we deliberately used GdBNs without any fluorescent marker in our present study on the DSBs induction and repair, as well as in our earlier works on the cell survival [23, 31].
4. Methodology used to monitor DSBs damage induction and repair: detection of DSBs repair foci by confocal immunofluorescence microscopy currently represents the most sensitive method to detect DSBs and monitor their repair. However, one should keep in mind some limitations of this method. Recent evidence suggests that γH2AX (histone H2AX phosphorylation on Ser139) alone may not always correspond with DSBs. In addition to ATM-mediated phosphorylation, H2AX can be phosphorylated also by ATR kinase in response to single-stranded DNA formation [60–62], such as during the replication stress caused by the replication fork arrest. Further, DNA-PK mediates phosphorylation of H2AX in cells during the apoptotic DNA fragmentation [52, 80]. Finally, γH2AX staining independent of DNA DSBs formation but related to nucleotide excision repair has previously been observed with primary human fibroblasts after UV irradiation [81]. In addition, it may be sometimes difficult to discriminate between true γH2AX foci and the background noise, especially in short periods of time post-irradiation (about 2–15 min PI) that are most relevant when DSBs induction is concerned [82]. Hence, we used independent immunolabelling of two DSBs markers—γH2AX and 53BP1—in combination with high-resolution confocal microscopy [82]. According to our best knowledge, this approach increases the accuracy of the DSBs recognition [83–86], and has not been used in earlier studies.

Further experimental incompatibility between studies follow from the fact that some authors analyzed the DSBs foci induction early PI [22] but the others [10, 46] rather followed the repair process, assuming that only the unrepaired DNA damage would lead to cell death or at least prevent further cell division. Here, we studied both the formation of DSBs immediately (5 min) PI and the removal of these lesions in several periods of time PI, up to 24 h PI. Therefore, we significantly extend the DSBs experiments performed by Mowat et al. [22] and Miladi et al. [33] in several aspects.

## Conclusions

We demonstrate that GdBNs of parameters defined in this study are localized in the cell cytoplasm and are not genotoxic. In conditions where these NPs exert significant radioenhancing effect they affect neither the induction of DNA double strand breaks nor their repair kinetics and efficiency. While further studies are needed to shed more light on processes forcing the cells to die after the cytoplasmic damage, and on the role of NPs in these processes, we can reasonably hypothesize on the basis of our results that electron showers and ROS emitted by irradiated NPs accumulated in lysosomes can disintegrate these organelles. This could be accompanied by a massive release of degradation enzymes into the cytoplasm and consequently auto-digestion and death of the cell. New super-resolution ‘nanoscopy’ techniques [77, 87] open opportunities to test our hypothesis directly in future.
